# Imaging Brain Injury in Former National Football League Players

**DOI:** 10.1001/jamanetworkopen.2023.40580

**Published:** 2023-10-30

**Authors:** Leah H. Rubin, Yong Du, Shannon Eileen Sweeney, Riley O’Toole, Cykyra L. Thomas, Adeline G. Zandi, Laura K. Shinehouse, Mary Katherine Brosnan, Hwanhee Nam, Michael E. Burke, Samantha C. Bureau, Jessica J. Kilgore, Mark Yoon, Ana R. Soule, Wojciech G. Lesniak, Il Minn, Steven P. Rowe, Daniel P. Holt, Andrew W. Hall, William B. Mathews, Gwenn S. Smith, Christopher J. Nowinski, Michael Kassiou, Robert F. Dannals, Martin G. Pomper, Jennifer M. Coughlin

**Affiliations:** 1 Department of Neurology, Johns Hopkins Medical Institutions, Baltimore, Maryland; 2 Department of Psychiatry and Behavioral Sciences, Johns Hopkins Medical Institutions, Baltimore, Maryland; 3 Department of Epidemiology, Johns Hopkins Medical Institutions, Baltimore, Maryland; 4 Department of Molecular and Comparative Pathobiology, Johns Hopkins Medical Institutions, Baltimore, Maryland; 5 Department of Radiology and Radiological Science, Johns Hopkins Medical Institutions, Baltimore, Maryland; 6 Concussion Legacy Foundation, Boston, Massachusetts; 7 Alzheimer’s Disease and CTE Center, Boston University School of Medicine, Boston, Massachusetts; 8 School of Chemistry, University of Sydney, Sydney, New South Wales, Australia

## Abstract

**Importance:**

Pilot studies that involved early imaging of the 18 kDa translocator protein (TSPO) using positron emission tomography (PET) indicated high levels of TSPO in the brains of active or former National Football League (NFL) players. If validated further in larger studies, those findings may have implications for athletes involved in collision sport.

**Objective:**

To test for higher TSPO that marks brain injury and repair in a relatively large, unique cohort of former NFL players compared with former elite, noncollision sport athletes.

**Design, Setting, and Participants:**

This cross-sectional study used carbon 11–labeled *N*,*N*-diethyl-2-(4-methoxyphenyl)-5,7-dimethylpyrazolo[1,5-*a*]pyrimidine-3-acetamide positron emission tomography ([^11^C]DPA-713 PET) data from former NFL players within 12 years of last participation in the NFL and elite noncollision sport athletes from across the US. Participants were enrolled between April 2018 and February 2023.

**Main outcomes and measures:**

Regional [^11^C]DPA-713 total distribution volume from [^11^C]DPA-713 PET that is a measure of regional brain TSPO; regional brain volumes on magnetic resonance imaging; neuropsychological performance, including attention, executive function, and memory domains.

**Results:**

This study included 27 former NFL players and 27 former elite, noncollision sport athletes. Regional TSPO levels were higher in former NFL players compared with former elite, noncollision sport athletes (unstandardized β coefficient, 1.08; SE, 0.22; 95% CI, 0.65 to 1.52; *P* < .001). The magnitude of the group difference depended on region, with largest group differences in TSPO in cingulate and frontal cortices as well as hippocampus. Compared with noncollision sport athletes, former NFL players performed worse in learning (mean difference [MD], −0.70; 95% CI, −1.14 to −0.25; *P* = .003) and memory (MD, −0.77; 95% CI, −1.24 to −0.30; *P* = .002), with no correlation between total gray matter TSPO and these cognitive domains.

**Conclusions and relevance:**

In this cross-sectional study using [^11^C]DPA-713 PET, higher brain TSPO was found in former NFL players compared with noncollision sport athletes. This finding is consistent with neuroimmune activation even after cessation of NFL play. Future longitudinal [^11^C]DPA-713 PET and neuropsychological testing promises to inform whether neuroimmune-modulating therapy may be warranted.

## Key Points

**Question:**

Is high 18 kDa translocator protein (TSPO) that marks brain injury and repair detected in the brains of a large group of young, former American National Football League (NFL) players?

**Findings:**

In this large cross-sectional study of 54 athletes, [^11^C]DPA-713 positron emission tomography imaging revealed high brain TSPO in former NFL players compared with elite, noncollision sport athletes, consistent with high TSPO after cessation of NFL play. The NFL players also had lower performance in learning and memory.

**Meaning:**

These findings suggest that because brain injury with cognitive impairment was found in these relatively young, former NFL players, further tracking of TSPO levels in relation to neuropsychological performance over time is needed to understand whether these signs persist, progress, and/or warrant neuroimmune-modulating interventions.

## Teaser

This cross-sectional study examines the amounts of 18 kDa translocator protein with [^11^C]DPA-713 positron emission tomography imaging in the brains of former National Football League players and noncollision sport athletes.

## Introduction

Sports-related, repeated brain injury is associated with varied neurologic and psychiatric sequelae.^1^ The observed association between repeated, mild traumatic brain injury (mTBI) and cognitive impairment may be mediated by microglia, the brain’s immune cells.^2,3,4,5,6^ Tracking the microglial response to brain injury is possible using positron emission tomography (PET) with a radiotracer targeting the translocator protein 18 kDa (TSPO).^7^ TSPO, also known as the peripheral benzodiazepine receptor, is a mitochondrial protein that has been pursued as a diagnostic imaging marker for the detection of inflammatory signaling, and is a possible target for anti-inflammatory or neuroprotective therapy.^8^ In health, TSPO is expressed in relatively low levels by microglia and astrocytes across the brain. After brain insult, TSPO expression is highly increased by activated microglia as part of the neuroinflammatory response. Using PET with TSPO-targeting radiotracers, such as carbon 11–labeled ([^11^C])–(*R*)-PK11195 or [^11^C]PBR28, high TSPO has been found in the brains of individuals 6 months after a traumatic brain injury,^9,10^ with evidence of high TSPO for up to 17 years after a moderate to severe brain insult.^11^ Further understanding of the microglial response to repeated mTBI in collision sport promises to inform future guidelines for game safety and clinical practices to promote brain healing.

Compared with control data, PET with the radiotracer [^11^C] *N*,*N*-diethyl-2-(4-methoxyphenyl)-5,7-dimethylpyrazolo[1,5-*a*]pyrimidine-3-acetamide (DPA-713) revealed high levels of TSPO across the brains of both elderly former National Football League (NFL) players^12^ and younger, active or former NFL players.^13^ However, each study was small in size, with just 9 to 10 former NFL players. Here we used [^11^C]DPA-713 PET in a larger, cross-sectional study of 27 former NFL players and 27 former elite, noncollision sport athletes. We hypothesized higher brain TSPO, marking microglial response to injury, in NFL players compared with noncollision sport athletes.

## Methods

The cross-sectional study was approved by the Johns Hopkins Institutional Review Board and Radiation Safety Committees and was conducted between April 2018 and February 2023. Each participant provided written, informed consent. This study followed the Strengthening the Reporting of Observational Studies in Epidemiology (STROBE) reporting guideline.

### Human Participants

The study included male individuals between ages of 23 to 50 years old who were assessed as either a (1) former NFL player within 12 years of last NFL play or (2) former noncollision sport athlete with at least 2 years of National Collegiate Athletic Association Division I, II, or III level play. The focus on former NFL players within 12 years of last NFL play was informed by the prior pilot finding of high TSPO in athletes within 12 years of last NFL play.^13^ NFL players were recruited through referral from author C.J.N. or word-of-mouth. Noncollision sport athletes were recruited through advertising in sports magazines with national readership.

A screening visit included laboratory testing and magnetic resonance imaging (MRI) of the brain. Participants were excluded if they had: (1) unstable health in the past year; (2) acute illness in the past month; (3) lack of English fluency; (4) recent (within 2 weeks) use of a medication or other substance with possible influence on [^11^C]DPA-713 binding (eg, anti-inflammatory medication, creatine^13^); (5) nicotine dependence or active substance misuse (cannabis use was permitted); or (6) contraindication to MRI or PET with an arterial line. After the screening visit, participants were excluded further if they were found to have the low affinity binding genotype (T/T) on rs6971 single nucleotide variant genotyping (*TSPO* genotyping).^12^ The rs6971 missense variant in the TSPO gene results in 3 distinct genotypes due to an amino acid substitution from alanine (ALA) to threonine (THR). These genotypes are C/C (ALA/ALA), C/T (ALA/THR), and T/T (THR/THR).

### Clinical and Cognitive Assessments

A clinical research interview was conducted by a study team clinician. Race was self-reported using options of Asian, Black, Hispanic/Latinx, White, or other (ie, any other race reported outside the other categories listed and included the opportunity to self-report mixed race), and collected to describe the population. History of sport participation and head health was also conducted by a study team clinician. Details about past concussion, as defined by the international Concussion in Sport Group since 2000,^14^ were self-reported. Cognition was assessed using a battery of neuropsychological tests across 9 cognitive domains (eTable 1 in Supplement 1), tailored to accommodate the relatively young, college-educated study population. Cognitive test scores were transformed into standardized *z* scores using data from the noncollision sport athletes. Mean *z* scores for each cognitive domain were calculated. Domain-specific *z* scores were averaged to create a mean global *z* score and a dispersion score, the latter herein referred to as intra-individual variability (IIV) across neuropsychological testing.^15^

### Imaging

Each participant completed one brain MRI at 3 Tesla and one [^11^C]DPA-713 PET scan (eAppendix 1 in Supplement 1). [^11^C]DPA-713 was synthesized in compliance with standard good manufacturing practice using published methods,^16^ and was delivered by intravenous slow push at the beginning of 90-minute of continuous emission data collection.^12^ Arterial blood sampling was performed to measure plasma time-activity and radiolabeled metabolite fraction (eAppendix 2 in Supplement 1).

### PET Kinetic Analysis

PMOD version 3.7 (PMOD Technologies) was used to generate the metabolite-corrected arterial input function, process image data, and perform kinetic analyses. PET data were rigidly transformed into MRI space.^12^ The binding outcome, total distribution volume (V_T_), was derived by applying Logan graphical analysis (t* = 30-minute)^17^ to each regional time-activity curve with the metabolite-corrected arterial input function.

### Statistical Analysis

Group differences in regional V_T_ values were examined using a single linear mixed model with repeated measures. Data were analyzed in SAS version 9.4 (SAS Institute). Variables in the model included group, an index variable for brain region, TSPO genotype (C/C genotype, high affinity binder; C/T genotype, mixed affinity binder), all 2-way interactions, and the 3-way interaction. Statistical significance was set at *P* < .05, and regional effect size (Cohen *d*) was calculated. Within the NFL players cohort, the association between [^11^C]DPA-713 gray matter (GM) V_T_ and domain-specific neuropsychological performance was assessed using partial correlations, with TSPO genotype as a covariate.

## Results

### Study Population

Twenty-seven NFL players (27 [100] males; mean [SD] age, 33.5 [3.9] years) and 27 noncollision sport athletes (27 [100] males; mean [SD] age, 32.4 [6.3] years) were eligible for the study, with an inclusive age range between 24 to 45 years. All the noncollision sport athletes were swimmers (27 [100%]). In addition to male sex and age, the groups were matched in reading skill (Word Reading Subtest-raw: mean [SD], NFL players, 63.9 [3.9] and noncollision sport athletes, 64.8 [3.3]) (Table). NFL players had a higher mean (SD) body mass index (calculated as weight in kilograms divided by height in meters squared; 30.4 [4.7]) than noncollision sport athletes (25.6 [3.9]). The NFL group consisted of 11 Black athletes (41%) and 16 White athletes (59%), and the noncollision sport group consisted of 2 Asian athletes (7%), 1 Black athlete (4%), and 24 White athletes (89%). The NFL group ceased NFL play within 1 to 12 years prior to participation. Other details about NFL play are listed in the Table.

**Table.** Demographic and Cognitive Characteristics of the Study Population

Characteristics	NFL players (n = 27)	Noncollision sport athletes (n = 27)	Mean difference (95% CI)	*P* value
Demographics				
Age, mean (SD)	33.5 (3.9)	32.4 (6.3)	NA	.45
WRAT, Word Reading Subtest-raw, mean (SD)	63.9 (3.9)	64.8 (3.3)	NA	.34
Race, No. (%)				
Asian	0	2 (7)	NA	.003
Black	11 (41)	1 (4)	NA	
White	16 (59)	24 (89)	NA	
BMI, mean (SD)	30.4 (4.7)	25.6 (3.9)	NA	<.001
TSPO (rs6971) genotype, No. (%)				
C/C (high affinity binder)	19 (70)	13 (48)	NA	.10
C/T (mixed affinity binder)	8 (30)	14 (52)	NA	
Cannabis use, No. (%)				
Never	5 (19)	9 (33)	NA	<.001
Occasional current	6 (22)	2 (7)	NA	
Daily-to-weekly current	12 (44)	1 (4)	NA	
Remote past use	4 (15)	15 (56)	NA	
American football experience				
Years played in the NFL, mean (SD)	6.0 (2.8)	NA	NA	NA
Years since last reported mTBI, median (IQR)	6 (4)	NA	NA	NA
Age at worst reported mTBI, mean (SD)	23.6 (3.8)	NA	NA	NA
Age at first reported mTBI, mean (SD)	15.6 (4.7)	NA	NA	NA
Player position, No. (%)				
Lineman, tight end	14 (26)	NA	NA	NA
Linebacker, running back	9 (17)	NA	NA	NA
Wide receiver, defensive back, quarterback	2 (4)	NA	NA	NA
Kicker, punter	2 (4)	NA	NA	NA
Cognitive test performance, mean (SE)^a^				
Mean composite *z* scores				
Global	−0.25 (0.58)	0 (0.25)	−0.25 (−0.50 to −0.01)	.04
Processing speed	−0.26 (0.83)	0 (0.46)	−0.26 (−0.63 to 0.10)	.15
Attention	−0.12 (0.73)	0 (0.83)	−0.12 (−0.55 to 0.30)	.57
Executive function	0 (0.80)	0 (0.30)	0.003 (−0.33 to 0.33)	.99
Verbal fluency	−0.04 (1.20)	0 (0.88)	−0.04 (−0.61 to 0.54)	.90
Visuoperception	−0.40 (1.04)	0 (0.71)	−0.40 (−0.89 to 0.09)	.11
Naming	−0.17 (1.34)	0 (1.00)	−0.17 (−0.82 to 0.48)	.60
Learning	−0.70 (0.93)	0 (0.68)	−0.70 (−1.14 to −0.25)	.003
Memory	−0.77 (0.98)	0 (0.72)	−0.77 (−1.24 to −0.30)	.002
Recognition	−0.25 (1.10)	0.01 (0.85)	−0.26 (−0.80 to 0.28)	.34
Intra-individual variability, mean (SE)	1.16 (0.31)	0.96 (0.20)	0.20 (0.06 to 0.34)	.006

Abbreviations: BMI, body mass index (calculated as weight in kilograms divided by height in meters squared); mTBI, mild traumatic brain injury; NA, not applicable; NFL, National Football League; SE, standard error; TSPO, 18 kDa translocator protein; WRAT, Wide Range Achievement Test.

^a^ Composite *z* scores, normed to the controls.

### Neuropsychological Assessment

Compared with noncollision sport athletes, NFL players performed worse globally (mean [SE], 0 [0.25] vs −0.25 [0.58]) and, specifically, in learning (mean [SE], 0 [0.68] vs −0.70 [0.93]) and memory (mean [SE], 0 [0.72] vs −0.77 [0.98]) (Table). The groups did not differ in mean performance across other domains. However, NFL players demonstrated greater IIV across neuropsychological testing compared with noncollision sport athletes.

### MRI and [^11^C]DPA-713 PET Imaging

**Figure 1.**
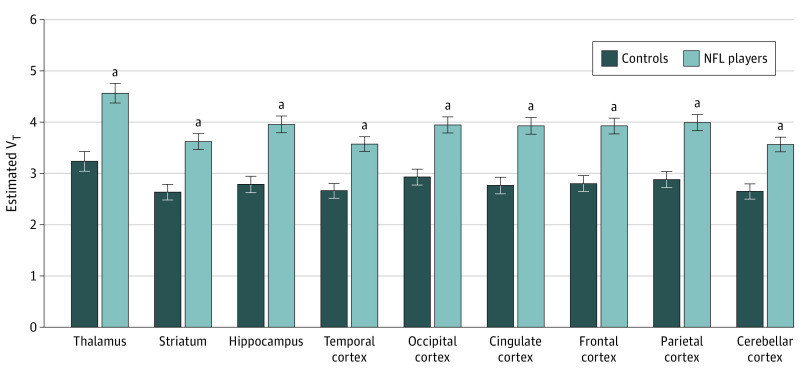
Comparison of Regional 18 kDa Translocator Protein (TSPO) Levels Measured With [^11^C]DPA-713 Positron Emission Tomography Between Former NFL Players and Noncollision Sport Athletes (Controls) Estimated V_T_ is in units of mL·cm^-3^ adjusted for TSPO rs6971 SNP genotype (C/C, C/T). Error bars reflect standard error. [^11^C]DPA-713 indicates carbon 11–labeled *N*,*N*-diethyl-2-(4-methoxyphenyl)-5,7-dimethylpyrazolo[1,5-*a*]pyrimidine-3-acetamide; NFL, National Football League; V_T_, total distribution volume. ^a^*P* < .001.

**Figure 2.**
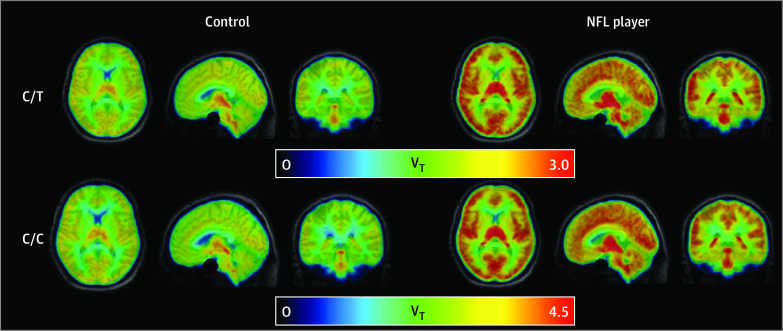
18 kDa Translocator Protein (TSPO) in Brains of Former National Football League (NFL) Players Compared With Former Noncollision Sport Athletes (Controls) Mean parametric maps of carbon 11–labeled *N*,*N*-diethyl-2-(4-methoxyphenyl)-5,7-dimethylpyrazolo[1,5-*a*]pyrimidine-3-acetamide binding (V_T_) are presented in 3 views for each group (NFL, control) within each TSPO genotype (C/C, C/T). V_T_ indicates total distribution volume and is in units of mL·cm^−3^.

There was no group difference in intracranial volume-adjusted region of interest (ROI) volumes (eTable 2 in Supplement 1). In genotype-adjusted analyses, [^11^C]DPA-713 V_T_ was higher in NFL players compared with noncollision sport athletes (unstandardized β coefficient, 1.08; SE, 0.22; 95% CI, 0.65-1.52; *P* < .001). The magnitude of the group difference in V_T_ depended on region, with largest group differences in cingulate cortex (β, 1.16; SE, 0.23; 95% CI, 0.91-1.86), frontal cortex (β, 1.12; SE, 0.22; 95% CI, 0.68-1.57), and hippocampus (β, 1.17; SE, 0.23; 95% CI, 0.71-1.62) (*P* = .002) (Figure 1). Mean parametric maps of V_T_ by group and TSPO genotype are presented in Figure 2. The 3-way interaction between group, ROI, and TSPO genotype was not significant (eTable 3 in Supplement 1). Within NFL players, there were no associations between [^11^C]DPA-713 GM V_T_ and either cognitive performance in any domain or IIV across neuropsychological testing.

## Discussion

In this cross-sectional PET study, higher levels of TSPO that mark brain injury and repair were found in former NFL players compared with former noncollision sport athletes. The most robust group differences in regional TSPO levels occurred in cingulate cortex, frontal cortex, and hippocampus, which is largely consistent with findings in smaller studies.^12,13^ Lower performance on tests of learning and memory, but not recognition, were found in NFL players relative to noncollision sport athletes. That result suggests a subcortical pattern of impaired encoding and memory retrieval that spares memory storage and retention, which contrasts the pattern in amnestic mild cognitive impairment.^18^ We also found greater IIV across neuropsychological testing in the NFL players, which may portend future cognitive decline.^15^

This study had limitations. While the findings suggest past brain injury in professional American football players, we acknowledge that TSPO is a marker of both brain injury and repair.^19^ These cross-sectional findings may reflect an ongoing reparative process that will resolve in the brains of former athletes with more time away from repeated brain injury. We also acknowledge that TSPO is expressed by cell types other than microglia. Microglia are the likely source of high TSPO in the brain after repeated mTBI, and yet TSPO can also be expressed by reactive astrocytes, infiltrating macrophages, and endothelial cells.^20^

[^11^C]DPA-713 PET has demonstrated clinical research use in detecting the high TSPO marker of brain injury in former NFL players. Those data suggest a persistence in neuroimmune activation due to the 1- to 12-year gap since they last played in the NFL, which may be tested using [^11^C]DPA-713 PET in longitudinal design. Further study of TSPO biology after mTBI is also needed.^20^

## Conclusion

This cross-sectional study found that higher TSPO may be present in former NFL athletes compared with noncollision sport athletes, which is consistent with neuroimmune activation even after cessation of NFL play. Longitudinal tracking of both TSPO and neuropsychological performance in former NFL players may inform whether neuroimmune therapy to promote brain healing is warranted.

## Notes

Supplement 1. **eAppendix 1.** MRI Acquisition and Regions of Interest **eAppendix 2.** [^11^C]DPA-713 and Metabolite Measures **eTable 1.** Neuropsychological Assessments **eTable 2.** Comparison of Regional Brain Volumes Adjusted for Intracranial Volume Between Former NFL Players and Former Noncollision Sport Athletes (Controls) **eTable 3.** Mean [^11^C]DPA-713 Binding (V_T_) Values for Each Region of Interest From Former NFL Players (NFL) and Former Noncollision Sport Athletes (Controls), Grouped by TSPO Genotype **eTable 4.** Effect of Demographic- and NFL Play-Related Characteristics on [^11^C]DPA-713 Binding (V_T_) in Gray Matter, Controlling for TSPO Genotype
eReferences.

Supplement 2.
Data Sharing Statement
