# Supplementary material for: Imaging Brain Injury in Former National Football League Players
Source: JAMA Netw Open. 2023 Oct 30;6(10):e2340580. doi: 10.1001/jamanetworkopen.2023.40580 (PMC10616723; doi:10.1001/jamanetworkopen.2023.40580)
Supplement: Supplement 1. — eAppendix 1. MRI Acquisition and Regions of Interest eAppendix 2. [11C]DPA-713 and Metabolite Measures eTable 1. Neuropsychological Assessments eTable 2. Comparison of Regional Brain Volumes Adjusted for Intracranial Volume Between Former NFL Players and Former Noncollision Sport Athletes (Controls) eTable 3. Mean [11C]DPA-713 Binding (VT) Values for Each Region of Interest From Former NFL Players (NFL) and Former Noncollision Sport Athletes (Controls), Grouped by TSPO Genotype eTable 4. Effect of Demographic- and NFL Play-Related Characteristics on [11C]DPA-713 Binding (VT) in Gray Matter, Controlling for TSPO Genotype eReferences. [file jamanetwopen-e2340580-s001.pdf]

Supplemental Online Content

Rubin LH, Du Y, Sweeney SE, et al. Imaging brain injury in former National Football League players. *JAMA Netw Open*. 2023;6(10):e2340580. doi:10.1001/jamanetworkopen.2023.40580

eAppendix 1. MRI Acquisition and Regions of Interest
eAppendix 2. [11C]DPA-713 and Metabolite Measures
eTable 1. Neuropsychological Assessments
eTable 2. Comparison of Regional Brain Volumes Adjusted for Intracranial Volume Between Former NFL Players and Former Noncollision Sport Athletes (Controls)
eTable 3. Mean [11C]DPA-713 Binding (V_T) Values for Each Region of Interest From Former NFL Players (NFL) and Former Noncollision Sport Athletes (Controls), Grouped by TSPO Genotype
eTable 4. Effect of Demographic- and NFL Play-Related Characteristics on [11C]DPA-713 Binding (V_T) in Gray Matter, Controlling for TSPO Genotype
eReferences.

This supplemental material has been provided by the authors to give readers additional information about their work.

© 2023 Rubin LH et al. *JAMA Network Open*.

eAppendix 1. MRI Acquisition and Regions of Interest

Structural T1-weighted Magnetization-Prepared Rapid Gradient-Echo (MPRAGE) was acquired at 3 Tesla (0.75 x 0.75 x 0.8 mm voxel size) on either a Philips Ingenia CX dStream or a Phillips Elition scanner (Philips Healthcare, Amsterdam, Netherlands). MPRAGE data were processed using the FreeSurfer image analysis suite, version 5[1] to delineate nine regions of interest (ROIs): thalamus, striatum, hippocampus, as well as cerebellar, temporal, occipital, cingulate, frontal, and parietal cortices. Total cortical gray matter (GM) was defined to test for association between [^{11}C]DPA-713 GM binding and neuropsychological performance.

eAppendix 2. [^{11}C]DPA-713 and Metabolite measures

[^{11}C]DPA-713 was synthesized as previously described,[2] and delivered by intravenous injection at the beginning of emission data collection.[3] The mean molar activity of [^{11}C]DPA-713 was 304.2 ± 89.6 GBq per micromole (8,221 ± 2,421 mCi per micromole) at time of injection. The mean injected dose was 703.3 ± 13.2 MBq. Emission data were acquired on a brain-dedicated High Resolution Research Tomograph (Siemens Healthcare, Knoxville, TN), with a fitted thermoplastic facemask for head fixation. The 90 min list mode data were binned into 30 frames and reconstructed using the iterative ordered subsets expectation maximization algorithm.[3]

Quantification of the relative percent of [^{11}C]DPA-713 and its radiometabolites was carried out using blood samples collected at 5, 10, 20, 30, 45, 60 and 90 min after administration of [^{11}C]DPA-713. A reverse phase high performance liquid chromatography (RP-HPLC) system from Agilent Technologies (Santa Clara, CA) was used, consisting of a 1260 Infinity quaternary pump, column compartment module, and UV and Raytest GABI Star radiation detectors, controlled by OpenLab CDS EZChrom (A.01.04) software. The RP-HPLC system was calibrated using [^{11}C]DPA-713 and a non-radioactive analog of the radiotracer before blood plasma sample analysis. For detection of cold DPA-713, a 266 nm wavelength was used. The RP-HPLC system contained a manual Rheodyne injector, inline capture column packed with Phenomenex Strata-X 33µm polymeric sorbent, and a Waters 4.6 x 150 mm, 5 µm XBridge column. Blood plasma samples (0.2-2 mL depending on the time point) were loaded onto the injector and directed to the capture column and the detectors with mobile phase composed of deionized water and 1% acetonitrile (ACN) at 2 mL/min. An analytical mobile phase contained 50% aqueous 0.06 M ammonium formate and 50% ACN. After 2 min of elution, an analytical mobile phase was applied to elute [^{11}C]DPA-713 and hydrophobic radiometabolites on the capture column to the analytical column and detectors. Radiometabolite-corrected plasma time-activity curves (TACs) were derived using the relative percent of parent [^{11}C]DPA-713 time-profiles obtained using RP-HPLC to the total plasma TACs after linear interpolation using PMOD (v3.7, PMOD Technologies Ltd, Zurich, Switzerland).

eTable 1. Neuropsychological Assessments

Construct	Neuropsychological Test*
Estimated General Intellectual Functioning	Wide Range Achievement Test, Word Reading Subtest[4]
Processing Speed	Trail Making Test-Part A[5]; Stroop Trials 1 and 2[6]; WAIS-IV Coding subtest[7]
Attention	WAIS-IV Digit Span Forward[7]; California Verbal Learning Test, Second Edition-Trial 1[8]
Executive Function	Trail Making Test-Part B[5]; Stroop Trial (interference trial)[6]; Modified Wisconsin Card Sorting Test[9]; WAIS-IV Digit Span[7]
Verbal Fluency	DKEFS Verbal Fluency; Category fluency[10]
Visual-Spatial Function	Benton Judgement of Line Orientation[11]; Rey Complex Figure Test-copy[12]
Naming	Boston Naming Test[13]
Verbal and Visual-Spatial Learning, Memory, and Recognition	California Verbal Learning Test, Second Edition-total learning across trials, delayed free recall, recognition[8]; WMS logical memory-immediate and delayed free recall[14]; Rey Complex Figure Test-short and long delayed free recall[12]

*As part of the neuropsychological battery, each participant completed the Test of Memory Malingering (TOMM).[15] On the TOMM, the majority of participants scored a 50/50 with the exception of one former NFL player who had two errors on Trial 2 (score of 48/50) and two non-collision-sport athletes who each had one error on Trial 2 (each score of 49/50). All scores were therefore consistent with performance validity.

eTable 2. Comparison of regional brain volumes adjusted for intracranial volume between former NFL players and former non-collision-sport athletes (Controls).

Region of Interest	NFL players (n=27) M (SE)	Controls (n=27) M (SE)	β *(95% CI)*	*P*[a]
Thalamus	16.34 (0.22)	16.60 (0.22)	0.26 (-0.36, 0.88)	0.41
Striatum	22.15 (0.37)	22.03 (0.37)	0.12 (-0.93, 1.17)	0.82
Hippocampus	9.20 (0.15)	9.62 (0.15)	0.42 (0.12, 0.72)	0.06
Temporal Cortex	100.09 (1.91)	103.60 (1.91)	3.51 (-1.91, 8.89)	0.19
Occipital Cortex	49.19 (1.03)	49.97 (1.03)	0.78 (-2.14, 3.70)	0.60
Cingulate Cortex	22.57 (0.54)	22.71 (0.54)	0.14 (-1.39, 1.67)	0.86
Frontal Cortex	174.65 (3.16)	174.37 (3.16)	0.28 (-8.69, 9.25)	0.95
Parietal Cortex	114.60 (1.99)	118.54 (1.99)	3.94 (-1.71, 9.59)	0.17
Cerebellar Cortex	97.47 (1.84)	99.14 (1.84)	1.67 (-3.55, 6.89)	0.52

β=unstandardized beta coefficient which reflects the mean difference between NFL players and controls in each region of interest. CI=confidence interval; [a]*P*-values for group differences from a single linear mixed model with repeated measures. Adjusted regional volumes are listed as M=mean (SE=standard error) and presented in mm^3.

eTable 3. Mean [^{11}C]DPA-713 binding (V_T) values for each region of interest from former NFL players (NFL) and former non-collision-sport athletes (Controls), grouped by TSPO genotype.

	TSPO genotype							
	C/C				C/T			
**Region of Interest**	**NFL (N=19) M (SE)**	**Controls (N=13) M (SE)**	**β (95% CI)**	***P*-value**	**NFL (N=8) M (SE)**	**Controls (N=14) M (SE)**	**β (95% CI)**	***P*-value**
Thalamus	5.12 (0.22)	3.96 (0.27)	1.16 (0.44, 1.88)	0.002	3.24 (0.35)	2.56 (0.26)	0.68 (-0.23, 1.59)	0.13
Striatum	4.05 (0.18)	3.19 (0.22)	0.86 (0.29, 1.43)	0.004	2.61 (0.28)	2.11 (0.21)	0.50 (-0.22, 1.22)	0.16
Hippocampus	4.45 (0.19)	3.39 (0.23)	1.06 (0.45, 1.66)	0.0008	2.77 (0.29)	2.22 (0.22)	0.55 (-0.21, 1.31)	0.14
Temporal Cortex	3.98 (0.17)	3.18 (0.20)	0.80 (0.26, 1.34)	0.004	2.60 (0.25)	2.14 (0.19)	0.46 (-0.21, 1.13)	0.16
Occipital Cortex	4.37 (0.18)	3.47 (0.22)	0.90 (0.30, 1.49)	0.003	2.94 (0.28)	2.42 (0.21)	0.52 (-0.22, 1.26)	0.15
Cingulate Cortex	4.41 (0.19)	3.30 (0.23)	1.11 (0.50, 1.72)	0.0005	2.78 (0.29)	2.27 (0.22)	0.51 (-0.26, 1.28)	0.16
Frontal Cortex	4.37 (0.18)	3.32 (0.22)	1.05 (0.46, 1.64)	0.0007	2.87 (0.28)	2.32 (0.21)	0.55 (-0.19, 1.29)	0.12
Parietal Cortex	4.42 (0.18)	3.40 (0.23)	1.02 (0.42, 1.62)	0.001	2.96 (0.29)	2.39 (0.22)	0.57 (-0.18, 1.32)	0.12
Cerebellar Cortex	4.00 (0.17)	3.17 (0.20)	0.83 (0.29, 1.37)	0.003	2.53 (0.26)	2.17 (0.19)	0.36 (-0.32, 1.04)	0.27

β=unstandardized beta coefficient which reflects the mean difference between NFL players and controls in each region of interest. CI=confidence interval; Regional [^{11}C]DPA-713 V_T values, listed as M=mean (SE=standard error), are presented for each cohort (National Football League [NFL] players vs. Controls) and grouped by TSPO genotype (C/C vs. C/T). V_T is in units of mL cm^{-3}. Estimated means are from a single linear mixed model with repeated measures. The variables in the model included: group, region, TSPO genotype, all two- and three-way interactions. Note that the three-way interaction between group, region, and TSPO genotype was not significant (P=0.52). Rather there was a significant group difference (P=0.002) whereby NFL players had higher V_T values overall compared to controls. Moreover, there was a significant two-way interaction between group and region such that the magnitude of the group difference differed across regions (P=0.002).

© 2023 Rubin LH et al. *JAMA Network Open*.

eTable 4. Effect of demographic- and NFL play-related characteristics on [^{11}C]DPA-713 binding (V_T) in gray matter, controlling for TSPO genotype.

	Estimate β (SE)	95% CI	*P* value
Age	0.01 (0.02)	-0.04, 0.06	0.61
Wide Range Achievement Test-raw score	-0.000 (0.03)	-0.07, 0.07	0.99
White (vs. not White)	0.34 (0.29)	-0.25, 0.93	0.25
Body mass index	0.02 (0.02)	-0.03, 0.07	0.50
Cannabis use (vs. never)			
Occasional current	-0.31 (0.58)	-1.51, 0.89	0.60
Daily-to-weekly current	0.02 (0.51)	-1.05, 1.07	0.98
Remote past use	-0.27 (0.64)	-1.61, 1.06	0.67
Years since cessation of NFL play	0.03 (0.07)	-0.13, 0.09	0.71
Years played in the NFL	-0.002 (0.06)	-0.13, 0.13	0.97
Years since last reported mTBI	0.03 (0.05)	-0.07, 0.14	0.49
Age at worst reported mTBI	0.07 (0.04)	-0.03, 0.17	0.15
Age at first reported mTBI	0.04 (0.04)	-0.04, 0.12	0.29
Player position (vs. kicker, punter)			
Lineman, tight end	0.11 (0.73)	-1.40, 1.62	0.87
Linebacker, running back	0.31 (0.76)	-1.27, 1.89	0.69
Wide receiver, defensive back, quarterback	0.31 (0.98)	-1.72, 2.35	0.75

β=unstandardized regression coefficient; CI=confidence interval; SE=standard error; mTBI=mild traumatic brain injury

eReferences

1. Fischl B, Salat DH, Busa E, et al. Whole brain segmentation: automated labeling of neuroanatomical structures in the human brain. *Neuron*. Jan 31 2002;33(3):341-55. doi:10.1016/s0896-6273(02)00569-x

2. Coughlin JM, Wang Y, Ambinder EB, et al. In vivo markers of inflammatory response in recent-onset schizophrenia: a combined study using [11C]DPA-713 PET and analysis of CSF and plasma. *Translational Psychiatry*. 2016/04/01 2016;6(4):e777-e777. doi:10.1038/tp.2016.40

3. Coughlin JM, Wang Y, Munro CA, et al. Neuroinflammation and brain atrophy in former NFL players: An in vivo multimodal imaging pilot study. *Neurobiology of disease*. Feb 2015;74:58-65. doi:10.1016/j.nbd.2014.10.019

4. Wilkinson GS, Robertson GJ. *Wide range achievement test 4*. Psychological Assessment Resources; 2006.

5. Reitan RM. The relation of the trail making test to organic brain damage. *Journal of consulting psychology*. Oct 1955;19(5):393-4.

6. Stroop JR. Studies of interference in serial verbal reactions. *Journal of experimental psychology*. 1935;18(6):643.

7. Wechsler D. *WAIS-IV: Wechsler Adult Intelligence Scale*. Pearson Assessment; 2008.

8. Delis DC, Wetter SR, Jacobson MW, et al. Recall discriminability: utility of a new CVLT-II measure in the differential diagnosis of dementia. *Journal of the International Neuropsychological Society*. 2005;11(6):708-15.

9. Nelson HE. A modified card sorting test sensitive to frontal lobe defects. *Cortex; a journal devoted to the study of the nervous system and behavior*. Dec 1976;12(4):313-24.

10. Delis DC, Kaplan E, Kramer JH. *Delis Kaplan Executive Function System (D-KEFS)*. Psychological Corporation; 2001.

11. Benton AL. *Benton Judgement of Line Orientation*. Psychological Assessment Resources; 1983.

12. Bennett-Levy J. Determinants of performance on the Rey-Osterrieth Complex Figure Test: an analysis, and a new technique for single-case assessment. *The British journal of clinical psychology / the British Psychological Society*. May 1984;23 (Pt 2):109-19.

13. Kaplan E, Goodglass H, Weintraub S. *Boston naming test*. Lea & Febiger; 1983.

14. Wechsler D. A standardized memory scale for clinical use. *The Journal of Psychology*. 1945;19(1):87-95.

15. Tombaugh T, ed. *Test of memory malingering*. Multi-Health Systems Inc; 1996.
