# Supplementary material for: Imaging Brain Injury in Former National Football League Players
Source: JAMA Netw Open. 2023 Oct 30;6(10):e2340580. doi: 10.1001/jamanetworkopen.2023.40580 (PMC10616723; doi:10.1001/jamanetworkopen.2023.40580)
Supplement: Supplement 2. — Data Sharing Statement [file jamanetwopen-e2340580-s002.pdf]

Data Sharing Statement

Rubin. Imaging Brain Injury in Former National Football League Players. *JAMA Netw Open*. Published October 30, 2023. doi:10.1001/jamanetworkopen.2023.40580

Data
Data available: Yes
Data types: Deidentified participant data
How to access data: jcoughl2@jhmi.edu
When available: With publication

Supporting Documents
Document types: None

Additional Information
Who can access the data: The data that support the findings of this study are available from the corresponding author on reasonable request.
Types of analyses: Data may be made available on request for any scientific purpose.
Mechanisms of data availability: Data may be made available after approval of a proposal and with signed data access agreement.
